# Medical Students’ Attitudes toward Abortion Education: Malaysian Perspective

**DOI:** 10.1371/journal.pone.0052116

**Published:** 2012-12-27

**Authors:** Nai-peng Tey, Siew-yong Yew, Wah-yun Low, Lela Su’ut, Prachi Renjhen, M. S. L. Huang, Wen-ting Tong, Siow-li Lai

**Affiliations:** 1 Faculty of Economics and Administration, University of Malaya, Kuala Lumpur, Malaysia; 2 Faculty of Medicine, University of Malaya, Kuala Lumpur, Malaysia; 3 Faculty of Medicine and Health Sciences, University Malaysia Sarawak (UNIMAS), Sarawak, Malaysia; 4 Department of Obstetrics and Gynaecology, Melaka Manipal Medical College, Melaka, Malaysia; 5 Faculty of Medicine and Health Sciences, Universiti Putra Malaysia, Selangor, Malaysia; Indiana University, United States of America

## Abstract

**Background:**

Abortion is a serious public health issue, and it poses high risks to the health and life of women. Yet safe abortion services are not readily available because few doctors are trained to provide such services. Many doctors are unaware of laws pertaining to abortion. This article reports survey findings on Malaysian medical students’ attitudes toward abortion education and presents a case for including abortion education in medical schools.

**Methods and Results:**

A survey on knowledge of and attitudes toward abortion among medical students was conducted in two public universities and a private university in Malaysia in 2011. A total of 1,060 students returned the completed questionnaires. The survey covered about 90% of medical students in Years 1, 3, and 5 in the three universities. About 90% of the students wanted more training on the general knowledge and legal aspects of abortion, and pre-and post-abortion counseling. Overall, 75.9% and 81.0% of the students were in favor of including in medical education the training on surgical abortion techniques and medical abortion, respectively. Only 2.4% and 1.7% were opposed to the inclusion of training of these two methods in the curriculum. The remaining respondents were neutral in their stand. Desire for more abortion education was associated with students’ pro-choice index, their intention to provide abortion services in future practice, and year of study. However, students’ attitudes toward abortion were not significantly associated with gender, type of university, or ethnicity.

**Conclusions:**

Most students wanted more training on abortion. Some students also expressed their intention to provide abortion counseling and services in their future practice. Their desire for more training on abortion should be taken into account in the new curriculum. Abortion education is an important step towards making available safe abortion services to enable women to exercise their reproductive rights.

## Introduction

The World Health Organization (WHO) estimated that there were about 42 million abortions globally in 2008, of which half were unsafe abortions. Almost all of the unsafe abortions were in developing countries. Unsafe or clandestine abortions were frequently performed by providers without the necessary skills and in an environment that did not conform to the minimum medical standards. In some instances, abortions were self-induced. Unsafe abortions imposed heavy economic and health burdens on women and society [1], [2].

Unsafe abortion rate is the number of unsafe abortions per 1,000 women aged 15–44 years in a year, and is used to measure the level of unsafe abortion in a population. Unsafe abortion ratio is the number of unsafe abortions per 100 live births (as a proxy for pregnancies) in a year, and is a measure of the likelihood that a pregnancy will end in unsafe abortion rather than in a live birth [1]. Globally, the unsafe abortion rate in 2008 was about 14 per 1,000 women aged 15–44, and for every 100 live births there were 16 unsafe abortions. The incidence of unsafe abortion varied widely across regions, countries and within countries. The highest observed unsafe abortion ratios were in developing countries with incomplete estimates [3]. The unsafe abortion rate and abortion ratio was relatively high in Southeast Asia, with an estimated rate of 22 per thousand women and 28 per 100 live births respectively [1].

Information on the incidence of abortion in Malaysia is not readily available. However, there are good reasons to believe that abortion is on the rise because the fertility level has been declining from 3.5 children per woman in 1985 to about 2.3 today, despite the stagnation of contraceptive prevalence rate at about 50% for all methods and 33% for modern methods since the mid 1980s [4].

The abortion law in Malaysia has become relatively liberal after it was amended in 1989. It allows abortion in order to preserve the physical and mental health of women, in addition to saving the life of pregnant woman which used to be the only legal basis for abortion [5]. Under the amended law, third-party authorization by a medical professional is still required. The public, including some medical professionals are unaware of this law. Due to the lack of knowledge of abortion law/procedures, and a poor understanding of women’s need, many doctors do not provide abortion services [6], [7]. Medical doctors also lack training on abortion counseling and service as medical schools in Malaysia have given little attention to abortion education [8].

To our knowledge, medical curriculum in Malaysian universities has been designed without incorporating students’ opinion. In Malaysia, apart from a study on medical students’ perception of biostatistics and epidemiology module [9], no study has been conducted to obtain student feedback on the curriculum. Studies on students’ perception on abortion education in North America found that students played an active role in advocating abortion education in medical schools [10]–[14]. A review of the literature shows that no such study has been done in Malaysia and other developing countries.

In view of the growing demand for safe abortion and the dearth of research on this topic, a survey was conducted to find out medical students’ knowledge of and attitudes toward abortion, including abortion education. It is hoped that the dissemination of the findings from this study will provide a case for the inclusion of more abortion education in the medical schools in Malaysia and other developing countries, as a first step towards making safe abortion more readily available to women.

## Materials and Methods

### Ethics Statement

The proposal to conduct the survey was endorsed by the Medical Ethics Committee, University of Malaya Medical Centre, which specifically approved this study in writing. Permission was sought from the deans of the three medical schools, and informed consent was obtained from all respondents before the questionnaires were administered.

### Study Setting

Malaysia is a multi-ethnic country in Southeast Asia. Out of a total population of 28.3 million in 2010, 91.8% were Malaysian citizens and 8.2% were non-citizens. Among the citizens, the Malays made up 55.5%, Chinese 25.6%, Indians 7.3%, other ethnic groups 13.5% [15]. Today about 71% of the population lives in the urban areas, as compared to 28% in 1970. Malaysia has achieved remarkable socio-economic progress. It is classified as an upper middle income country by the World Bank, and a high human development index (HDI) country by the United Nations. The gross enrolment rate for primary, secondary and tertiary education is about 95%, 70% and 38% respectively [16]. A report of the Ministry of Higher Education put the total enrolment in public and private institutions of higher learning at 921,979 in 2009, with 202,203 graduating in that year [17]. In Malaysia, medical education is a 5-year undergraduate program. Upon completing the 5-year program, students are awarded the Bachelor of Medicine, Bachelor of Surgery (MBBS). Some proceed to pursue post graduate courses with specialization in a particular field. Currently, there are 33 medical schools in Malaysia, and around 4,000 medical students are expected to graduate with MBBS degree annually.

### Study Design and Data Collection

The survey was planned to cover two public universities and two private universities out of a total of 33 medical schools in Malaysia. The sampling design for this survey used a combination of stratified random sampling and single stage cluster sampling. The universities were stratified according to public and private universities, and two universities were randomly selected from each stratum. Under the one-stage sampling scheme, all Years 1, 3 and 5 medical students in the selected universities would be interviewed. The management of one private university did not give permission for the survey to be conducted, and no replacement was made. The prerequisites for admission of students are rather similar for all the universities as the entry criteria are being regulated by the Ministry of Higher Education. Hence, students from the three universities would be fairly representative of the study population.

The survey was carried out from March through May 2011 to cover all the Years 1, 3 and 5 medical students in the two selected public universities. However, the survey in the private university was confined to all Years 3 and 5 medical students, as Year 1 students were attending classes in a foreign campus. Research team members explained the objectives of the survey before distributing the questionnaires to the students for self administration during lecture sessions of core courses. A total of 1,060 students returned the questionnaires, representing a response rate of about 90%. Of the 1,060 students who returned the completed questionnaire, 69 did not answer questions pertaining to abortion education or pro-choice or intention to provide abortion services, and were excluded from the analysis.

### The Survey Instrument

The questionnaire was designed in consultation with key personnel from the Ministry of Health, the Reproductive Rights Advocacy Alliance of Malaysia (RRAAM) and the universities, to collect data on students’ knowledge of and attitudes toward induced abortion, and related reproductive health services including contraceptive use. The draft questionnaire was pre-tested on 30 Year 2 medical students in one of the public universities selected for the main survey.

The questionnaire consisted of 26 items. These items included students’ knowledge of abortion and reproductive health, attitudes toward abortion and abortion education (general knowledge, surgical and medical abortion, legal aspects of abortion, and pre- and post-abortion counseling), type of university (public and private), year of study, gender, ethnicity, and intention to provide abortion services in future practice. The questionnaire also included agreement or disagreement on 12 Likert scale statements on various conditions for abortion, such as on the grounds of rape, risk to women’s health and life, their rights and socio-economic considerations, from 1 for those who disagreed strongly (not pro-choice) to 5 for those who agreed strongly (pro-choice).

### Statistical Analysis

All data were entered into the computer and checked for data entry errors, missing data and inconsistencies. Data were analyzed using SPSS for Windows version 19. A pro-choice index was created by summing the responses to the 12 statements, with a Cronbach’s Alpha value of 0.8581. The index was further regrouped into 3 main categories: below 29, 29–36, and 37+. Respondent’s feedback to each of the six questions relating to their intention to provide abortion counseling, referrals and services were recoded as 2 if the answer was “Yes”, 1 if “Uncertain” and 0 if the answer was “No”, and the scores were added to form an index on willingness or intention to provide abortion services in future practice, with a Cronbach’s Alpha value of 0.7548. Generally a Cronbach’s Alpha value of between 0.7–0.8 indicates internal consistency for a reliable scale [18].

Frequency tables were run to show the distribution of the sample according to selected variables, and students’ attitudes toward various types of abortion education. Cross-tabulations were run to compare the proportion of students agreeing or disagreeing with abortion education across the categories of selected variables. Chi-square tests were used to test for significant association between the dependent and independent variables.

Because the independent variables may have confounding effects on students’ attitudes toward abortion education, multiple logistic regressions were performed to assess the net effects of selected variables on students’ opinions on four types of abortion education. The odds ratio from the logistic regression measures the probability of an event occurring (e.g. student agreeing to more abortion education) with the probability of an event not occurring [p/(1−p)], where p is the probability of an event occurring [19].

## Results

Of the 991 students in this analysis, exactly two thirds were from the public universities. A quarter of the respondents were Year 1 students, 32% were Year 3 students and 43% were Year 5 students. Sixty three percent were female. The sample comprised 46.9% Malays, 37.6% Chinese, 12.0% Indians, and 3.5% others (Table 1). The ethnic distribution of the respondents corresponded closely to the ethnic composition in these medical schools. There was almost equal number of students according to the three categories of pro-choice index. About half of the students had a score of 6 to 9 on their intention to provide abortion services in future practice, while about one quarter each had a score of below 6, and 10 and above respectively (Table 1).

**Table 1:** Distribution of respondents by selected variables.

	Frequency	Percent
Total	991	100.0
**Type of university**		
Public	661	66.7
Private	330	33.3
**Year of study**		
Year 1	249	25.1
Year 3	317	32.0
Year 5	425	42.9
**Gender**		
Male	363	36.6
Female	628	63.4
**Ethnicity**		
Malays	465	46.9
Chinese	372	37.6
Indians	119	12.0
Others	35	3.5
**Pro-choice index**		
Below 29	310	31.3
29–36	343	34.6
37+	338	34.1
**Intention to provide abortion service**		
Less than 6	240	24.2
6 to 9	509	51.4
10+	242	24.4

The majority of students (about 90% or more) agreed that there should be more training in general knowledge and legal aspects of abortion, and pre- and post-abortion counseling. However, 21.7% and 17.3% were neutral on the need for more training in surgical abortion techniques and medical abortion respectively. Only a small number of students disagreed with having more training on the various types of abortion education in the medical curriculum (Table 2).

**Table 2:** Students’ opinions on the inclusion of various types of abortion education into the medical curriculum (n = 991).

Types of abortion education	Strongly agree	Agree	Neutral	Disagree	Strongly disagree
General knowledge on abortion	47.6	45.2	6.8	0.2	0.2
Surgical abortion techniques	32.0	43.9	21.7	1.7	0.7
Medical abortion	34.0	47.0	17.3	1.2	0.5
Legal aspects of abortion	47.8	41.1	9.1	1.2	0.8
Pre- and post-abortion counseling	44.3	43.0	11.0	1.1	0.6

Male and female students from both public and private universities did not differ significantly in their opinion on any type of abortion education (Table 3). However, year of study was significantly associated with students’ attitudes toward more training on surgical and medical abortion (p<0.05) and the legal aspect of abortion (p<0.01). Year 5 students were much more likely than their juniors, especially Year 1 students, to want more training on abortion education. Senior students tended to have better knowledge on various aspects, including the curriculum, as compared to their juniors. This is consistent with finding of a study among medical students in the University of British Columbia, where senior students were found to be more knowledgeable than their juniors [14]. Being aware of the lack of abortion education through the years, it is not surprising that senior students were more likely to express a desire for wanting more training in abortion. There was no significant ethnic differential in the attitudes toward more training in general knowledge, surgical and medical abortion and the legal aspect. However, the Chinese and Indians were more likely than the Malays to want more training on pre- and post-abortion counseling (p<0.05). Pro-choice students were much more likely to want more training on surgical and medical abortion, and pre-and post-abortion counseling, as compared to those who were not pro-choice (p<0.01). As expected, the desire for abortion education was higher among students who expressed an intention to provide abortion service in future practice than those who had no intention to provide such service (p<0.01).

**Table 3:** Percent of respondents agreeing to the inclusion of the various types of abortion education in the curriculum by selected variables.

	General knowledge on abortion	Surgical abortion techniques	Medical abortion	Legal aspects of abortion	Pre- and post-abortion counseling
**Type of university**					
Public	92.0	75.3	79.6	87.7	86.1
Private	94.5	77.0	83.6	91.2	89.4
					
**Year of study**					
Year 1	89.6	72.7	75.9	81.5	83.5
Year 3	93.4	72.9	81.1	91.2**	87.1
Year 5	94.4	80.0	83.8*	91.5**	89.4
					
**Gender**					
Male	91.7	75.2	81.3	89.3	87.6
Female	93.5	76.3	80.7	88.7	86.9
					
**Ethnicity**					
Malays	90.8	74.0	78.5	88.2	83.9
Chinese	94.6	79.3	84.7	90.9	90.9*
Indians	94.1	76.5	79.8	85.7	88.2
Others	97.1	62.9	77.1	88.6	88.6
					
**Pro-choice index**					
Below 29	91.9	69.0	73.2	89.4	82.3
29–36	93.0	74.3	81.6*	88.6	88.6
37+	93.5	83.7**	87.3**	88.8	90.2**
					
**Intention to provide abortion service**					
Less than 6	90.8	64.6	68.3	85.4	77.1
6 to 9	92.9	76.2**	82.7**	88.2	88.2**
10+	94.6	86.4**	89.7**	93.8*	95.0**
					

Note: * statistically significant; p<0.05;

** p<0.01 as compared to the group with the lowest value. Differences of all other pairwise comparisons are not statistically significant except * between pro-choice 29–36 and 37+ on surgical abortion techniques and intention 6 to 9 and 10+ on counseling; and ** between intention 6 to 9 and 10+ on surgical abortion techniques.

In Table 4, an odds ratio of 0.86 indicates that the odds of students from private university wanting more training on surgical abortion techniques was 14% (1–0.86) lower than those from the public universities, the reference group. On the other hand, the odds of students from private university wanting more training on medical abortion was 1.09 times (or 9%) higher than those from public universities. However, difference in the opinion on the various types of abortion education between students from public and private universities was not statistically significant. Year 3 and Year 5 students were about 2.4 times and 2.5 times more likely than Year 1 students to want more training on the legal aspect of abortion, and the differences were statistically significant, as the confidence interval did not contain “1”. Malay students were more likely to want more training on surgical abortion techniques as compared to the other ethnic groups. On the other hand, Chinese students were more likely to want more training on the legal aspects of abortion and pre- and post-abortion counseling. However, the ethnic differentials in student attitudes toward more training on various types of abortion education were not statistically significant. Controlling for other variables in the model, pro-choice students and those who would provide abortion services in future practice were much more likely to want more training in all aspects of abortion education, as compared to those who were not pro-choice and would not provide abortion service in their future practice.

**Table 4:** Logistic regressions on students’ desire for the various types of abortion education.

	Surgical abortion techniques		Medical abortion		Legal aspects of abortion		Pre- and post- abortion counseling	
	Odds ratio	95% CI	Odds ratio	95% CI	Odds ratio	95% CI	Odds ratio	95% CI
**Type of university**								
Public (Reference)	1.00		1.00		1.00		1.00	
Private	0.86	(0.60, 1.25)	1.09	(0.72, 1.65)	1.08	(0.63, 1.87)	1.16	(0.71, 1.88)
**Year of study**								
Year 1 (Reference)	1.00		1.00		1.00		1.00	
Year 3	0.95	(0.64, 1.43)	1.19	(0.77, 1.84)	2.35**	(1.36, 4.05)	1.12	(0.67, 1.85)
Year 5	1.41	(0.92, 2.15)	1.34	(0.86, 2.10)	2.52**	(1.45, 4.39)	1.31	(0.78, 2.22)
**Ethnicity**								
Malays (Reference)	1.00		1.00		1.00		1.00	
Chinese	0.91	(0.63, 1.33)	1.01	(0.67, 1.52)	1.49	(0.89, 2.49)	1.53	(0.93, 2.50)
Indians	0.82	(0.49, 1.37)	0.69	(0.40, 1.21)	0.75	(0.39, 1.44)	1.13	(0.58, 2.20)
Others	0.57	(0.27, 1.20)	0.91	(0.39, 2.13)	1.14	(0.38, 3.42)	1.67	(0.56, 4.95)
**Pro-choice index**								
Below 29 (Reference)	1.00		1.00		1.00		1.00	
29–36	1.09	(0.75, 1.58)	1.28	(0.86, 1.92)	0.70	(0.41, 1.19)	1.11	(0.69, 1.80)
37+	1.71*	(1.08, 2.71)	1.69*	(1.03, 2.79)	0.53*	(0.28, 0.97)	0.89	(0.50, 1.59)
**Intention to provide abortion service**								
Less than 6 (Reference)	1.00		1.00		1.00		1.00	
6 to 9	1.53*	(1.07, 2.19)	1.88**	(1.28, 2.76)	1.25	(0.76, 2.04)	2.01**	(1.30, 3.11)
10+	2.72**	(1.65, 4.47)	3.01**	(1.75, 5.19)	2.70**	(1.34, 5.44)	5.07**	(2.49, 10.30)
Constant	1.98		1.53		3.67		2.18	

Note: Odds ratios and [95% confidence intervals] from logistic regression models predicting students’ desire to have more training on various aspects of abortion.

* and ** denote statistical significance at the 0.05 and 0.01 levels, respectively.

## Discussion

The transition from unsafe to safe abortion requires abortion training for service providers, and provision of services at the appropriate primary level health service delivery points, to ensure that women have access to these services instead of having to seek out services from untrained providers [20]. A study in Africa shows that with the introduction of medical abortion, thousands of women’s lives were saved [21]. Despite the call by the Edinburg Declaration of the World Medical Association (1998) [22] for medical schools to produce professionals who are able to understand the needs of the communities and to respond accordingly, medical schools in Malaysia and other developing countries do not offer sufficient training to equip students with the knowledge and skills necessary to counsel patients about abortion and to become abortion providers [14], [23].

In Malaysia, family planning services are not available to unmarried persons. With the trend towards delayed marriage [24], and more and more adolescents becoming sexually active, issues such as youth sexuality, unwanted pregnancies and baby abandonment have become serious social problems. Presumably, some women may have resorted to unsafe abortion.

The growing concern over adolescent sexual behavior led to the adoption of the National Adolescent Health Policy in 2001, and the National Reproductive Health and Social Education Policy in 2010. The stagnation of the contraceptive prevalence rate and high level of unmet need have also been highlighted at various forums. More innovative approaches, including abortion counseling and management, need to be adopted to complement the family planning efforts. Those responsible for undertaking the curriculum review may need to be more sensitive to the prevailing sentiments and concerns on the sexuality problems. New elements of abortion education such as counseling as a means to help prevent out-of-wedlock and unwanted pregnancies need to be introduced.

There will always be barriers to the inclusion of abortion education in the medical curriculum, even in developed countries [25], due to pressure from the anti-choice and pro-life movements. One way to circumvent the restrictions and rejection of abortion education is to incorporate abortion training into public and reproductive health education.

While greater contraceptive use can be expected to reduce induced abortion, as shown in a study by Bongaarts and Westoff [26], other studies showed no significant association between induced abortion and contraceptive use [27], [28]. The parallel rise in abortion and contraception in some countries has occurred because increased contraceptive use alone was unable to meet the growing need for fertility regulation in situations where fertility was falling rapidly [28]. Hence, there is a need to offer abortion services alongside family planning.

Non-government organizations (NGOs) are playing an important role in providing abortion information and counseling in Malaysia. The Federation of Reproductive Health Association Malaysia (FRHAM) has set a goal for universal recognition of women’s right to have access to safe abortion and a reduction in the incidence of unsafe abortion. FRHAM and its member associations aim to provide safe, sensitive, non-judgmental and affordable abortion-related services (including pre- and post-abortion counseling), with special attention to young women, the under-served and marginalized groups. These organizations also act as referral centers for post-abortion care, treatment of complications and contraceptive services [29]. In 2007, the Reproductive Rights Advocacy Alliance of Malaysia was formed as part of the Asia Safe Abortion Partnership, with the objective to inform, educate, and advocate on reproductive health issues, including abortion [30]. The missions and concerns of these NGOs point to the need to improve access to safe abortion services.

A survey on medical students in the United States found that 96% of the respondents indicated that abortion education was appropriate in the preclinical and clinical curricula. The same study also found that about three quarters of students who planned a career in Family Medicine and Obstetrics and Gynecology preferred the integration of abortion training into the residency curriculum [13]. Respondents in our survey were not asked about the appropriateness of abortion education in preclinical and clinical curricula. However, it is likely that medical students in Malaysia would share the same view with their American counterparts, as the level of acceptance of abortion training among medical students in Malaysia was as high as in the United States [10]–[13].

In planning the survey, special attention was given to the study design. Nevertheless, the study still has a number of limitations. The survey was conducted in only three universities. A selected private university did not give us the permission to conduct the survey, and it was not replaced with another university due to time constraint. Moreover, the survey did not collect information on the perceptions of normative beliefs within the family, among peers and society which would have an impact on attitudes toward abortion and abortion education. In a multi-ethnic country like Malaysia, ethnicity is an important variable in most socio-economic and attitudinal research, as the ethnic variable subsumes many cultural and religious dimensions. In some populations, religion may be an important variable in explaining people’s attitudes toward abortion. This variable was not included in the questionnaire because of the strong association between ethnicity and religion. In Malaysia, all Malays are Muslim, 84% of the Chinese are Buddhist, and 86% of the Indians are Hindu. It turned out that the ethnic variable which subsumes various socio-cultural and religious norms in Malaysia did not have any significant effect on the students’ attitudes toward abortion education. As more than 90% of students wanted at least some abortion education, any sampling bias is unlikely to change significantly the key finding that most medical students want more abortion education. Hence, the survey findings provide a strong case for abortion curriculum reform.

### Conclusion

Our survey showed that almost all medical students in both public and private universities were in favor of having more training in abortion. Moreover, about 80% of the medical students in this survey reported an intention to provide some forms of abortion services in their future practice. Clearly, there is a need to equip medical students with skills and knowledge to meet the increasing demand for safe abortion. Such a need was unanimously endorsed by participants of a national seminar held to discuss the findings from this survey and two other related surveys.

Abortion education can be incorporated into existing courses along with family planning and reproductive health modules to avoid any opposition that may arise. Curriculum reform to improve abortion education would result in improved reproductive health care for women, and to enable them to exercise their reproductive rights.
